# Neuroimaging biomarkers for predicting stroke outcomes: A systematic review

**DOI:** 10.1002/hsr2.2221

**Published:** 2024-07-01

**Authors:** Elizabeth Gaviria, Awab Hamid Eltayeb Hamid

**Affiliations:** ^1^ CES University Medellín Colombia; ^2^ Department of Neurology Nile University Khartoum Sudan

**Keywords:** neuroimaging, neuroimaging biomarkers, prediction of stroke outcomes, stroke outcome

## Abstract

**Background and Aims:**

Stroke is a prominent cause of long‐term adult impairment globally and a significant global health issue. Only 14% of stroke survivors achieve full recovery, while 25% to 50% require varying degrees of support, and over half become dependent. The aftermath of a stroke brings profound changes to an individual's life, with early choices significantly impacting their quality of life. This review aims to establish the efficacy of neuroimaging data in predicting long‐term outcomes and recovery rates following a stroke.

**Methods:**

A scientific literature search was conducted using the Centre of Reviews and Dissemination (CRD) criteria and PRISMA guidelines for a combined meta‐narrative and systematic quantitative review. The methodology involved a structured search in databases like PubMed and The Cochrane Library, following inclusion and exclusion criteria to identify relevant studies on neuroimaging biomarkers for stroke outcome prediction. Data collection utilized the Microsoft Edge Zotero plugin, with quality appraisal conducted via the CASP checklist. Studies published from 2010 to 2024, including observational, randomized control trials, case reports, and clinical trials. Non‐English and incomplete studies were excluded, resulting in the identification of 11 pertinent articles. Data extraction emphasized study methodologies, stroke conditions, clinical parameters, and biomarkers, aiming to provide a thorough literature overview and evaluate the significance of neuroimaging biomarkers in predicting stroke recovery outcomes.

**Results:**

The results of this systematic review indicate that integrating advanced neuroimaging methods with highly successful reperfusion therapies following a stroke facilitates the diagnosis of the condition and assists in improving neurological impairments resulting from stroke. These measures reduce the possibility of death and improve the treatment provided to stroke patients.

**Conclusion:**

These findings highlight the crucial role of neuroimaging in advancing our understanding of post‐stroke outcomes and improving patient care.

## Key points

- Stroke is a leading cause of long‐term impairment, with a high number of survivors dependent on others.
- This study emphasizes the potential of neuroimaging biomarkers in predicting stroke recovery outcomes, marking an advancement in personalized rehabilitation strategies.
- Neuroimaging biomarkers could revolutionize stroke rehabilitation, allowing for more tailored and effective treatment plans and improving patient outcomes.

## 1 INTRODUCTION

Stroke is a leading cause of long‐term adult disability and a significant global health issue. Although there was a significant statistical decrease in the average number of years for disability‐adjusted life and death, the overall number of stroke survivors increased dramatically between 1990 and 2013.^1^ Stroke is also a major risk factor for various dementias such as, vascular dementia and Alzheimer's dementia contributing to cognitive impairment. Vascular dementia is the result of the brain's blood supply being restricted by strokes or vascular lesions which subsequently cause cognitive deterioration.^2^ Vascular factors (e.g., hypertension and diabetes) that predispose people to stroke are also related to an increased risk of Alzheimer's.^3^ Only 14% of stroke survivors fully recover to resume their daily activities; 25% to 50% need some support, and around half become dependent on others.^4^ Since two‐thirds of all stroke cases occur in people under the age of 70, the total number of years of life with an impairment adjusted for ischemic stroke is substantial.^4^ The stroke cases among women are generally higher than those of men, which can be explained by the longer life span of women and their greater probability of having a stroke at the later stages of life.^5^ Various psychiatric, neurological, and cardiac conditions may result in stroke such as multiple sclerosis, ebsteins anomaly, takotsubo cardiomyopathy, fragile X syndrome, and so forth. Fragile X Syndrome patients may develop stroke due to vascular abnormalities associated with the condition.^6^ Takotsubo cardiomyopathy, also known as “broken heart syndrome,” is a stress‐induced heart condition that can lead to stroke due to transient heart dysfunction and hypercoagulability. Ebstein Anomaly may predispose individuals to stroke due to thromboembolic events, and paradoxical embolism.^7^

Early treatment decisions poststroke are essential to halt the progression of brain damage during ischemia and improve life quality. It should be based on therapeutic strategies that are practical and efficient in every particular situation.^8^ These decisions rely heavily on clinical data^8^ but also on imaging and laboratory tests. Magnetic resonance imaging (MRI) or computed tomography (CT) are currently used to confirm the diagnosis and regulation of large morphological lesions. However, prospective studies are necessary to demonstrate the validity of clinical evaluations based on data from morphologic imaging for predicting the effectiveness of therapy.^9^

Predicting the outcome of a stroke during the subacute phase, which lasts 1–3 weeks after the attack, is crucial for planning the patient's discharge, anticipating the potential consequences for adjustments to home and community support, and appropriately educating patients and their families about reasonable and achievable treatment and rehabilitation goals.^10^ Several systematic reviews have covered the connection between standardized measures and different elements of stroke outcome and recovery, such as the measurement of neurological impairments, functional outcomes, and standards of life.^11^, ^12^ Prognostic studies^12^ demonstrated that gender and the prevalence of factors associated with cardiovascular disease were not predictors of outcome; however, stroke severity, age, and motor weakness were identified as significant predictors. Several researchers have focused on designing specialized models for predicting recovery across various modalities, such as walking and motor functions, which are pivotal in rehabilitative treatments.^13^, ^14^ Evoked potential investigations are also helpful in selecting patients who can benefit from specific rehabilitative methods and evaluating the likelihood of functional recovery.^15^ It has also been demonstrated recently that recovery can be predicted using the causal classification of stroke.^16^

Because of the use of highly successful reperfusion treatments throughout the poststroke period and enhanced neuroimaging techniques, modern stroke patients experience better care and have a lower probability of death. These interventions contribute to the diagnosis of stroke and assist in the improvement of any neurological impairment caused by stroke. Stroke survivors, particularly those in underdeveloped nations, lack widespread access to diagnostic and treatment approaches. In addition, the apparent wide range in the neurological outcome following a stroke, from a mild, nondisabling form to a more severe type marked by lasting disability or even death, should be considered. As a result, using biomarkers has become an acceptable new technique for accurately predicting functional outcomes following stroke.^17^, ^18^, ^19^

The most current definition of biomarkers states that they are physiological features or biological substances that can be assessed objectively and reliably. They can be used as risk factors, pharmacologic responses to therapeutic intervention, or indicators of biological processes that are either normal or pathological. To be useful in clinical settings, a biomarker needs to have both high sensitivity and specificity,^20^, ^21^, ^22^ should be easily accessible, affordable, reproducible, noninvasive, and cost‐effective, giving the clinician information that can influence the therapeutic approach.^23^, ^24^

Modern neuroimaging methods are now extensively accessible for stroke evaluation. According to available data, models with clinical predictors can benefit from adding neuroimaging data by enhancing prediction accuracy in a clinically significant way.^25^ These models can potentially improve the validity of stroke outcome prediction measures, provide essential insights into the biology of stroke damage and its recovery, and be used as biomarkers to evaluate the effectiveness of therapy. However, more research is needed to determine which biomarkers are the most significant indicators of functional recovery following a stroke.^26^

This study reviews the updates of previous articles and discusses the contributions of different neuroimaging techniques to predicting the rate of recovery and the subsequent functional outcomes in patients following an ischemic stroke.^27^ It also contributes to the evaluation of rehabilitation therapy's efficacy in individual patients and clinical studies.

The primary aim of this review is to assemble compelling evidence supporting the hypothesis that neuroimaging may be beneficial for predicting long‐term outcomes after stroke. The focus is on examining the utility of neuroimaging rather than addressing whether the information obtained from acute stroke patients is helpful in selecting specific treatments.

## 2 METHODOLOGY

### 2.1 Design

The design of this investigation was comprehensive and theoretical. The Centre of Reviews and Dissemination criteria (CRD) from 2009 were followed by combining the meta‐narrative study and the systematic quantitative review. An initial search technique was performed, taking the inclusion and exclusion criteria into account. Based on the results, a search was conducted, and data extraction and synthesis were finished afterward. Ethics approval was not required for this systematic review.

### 2.2 Search method

The Recommended Reporting Criteria for Systematic Reviews and Meta‐Analysis (PRISMA) guidelines were used for this investigation. Search engines, including PubMed and The Cochrane Library databases, were used for the literature search, and articles published between 2010 and 2024 were reviewed. The search and selection options for the entire article are listed in Table 1. Articles with abstracts only and papers in all languages were added after a prescreening using the “Human studies only” criteria. Only publications that addressed the subject were included after closely reviewing each title and abstract.

**Table 1:** Search strategy used to find relevant articles.

Search number	Search terms	Results
#1	(Neuroimaging biomarkers) AND (prediction of stroke outcomes)	178
#2	Neuroimaging [MeSH Terms]	6
#3	(Neuroimaging for the prediction of recovery [Title/Abstract] AND long‐term outcome [Title/Abstract]) OR (neuroimaging biomarkers in stroke [Title/Abstract])	102

### 2.3 Inclusion criteria

The following are the criteria used to include studies:

1. Observational and randomized control trials, case reports, and clinical trials evaluated neuroimaging biomarkers for stroke outcome prediction.
2. Research addressing the use of neuroimaging to predict long‐term results and the extent of recovery.

### 2.4 Exclusion criteria

The excluded articles did not discuss the relationship between stroke outcomes and neuroimaging biomarkers. Translation issues resulted in the removal of papers not published in English. Articles relevant to the review topic but not containing the entire source material were also excluded.

### 2.5 Data collection method

The Microsoft Edge Zotero plugin was used to find the articles in the databases. The abstracts and titles were loaded, and the Zotero desktop was opened. After the data was gathered, tables were created on the Excel document. The information obtained from the combined research and results produced in this systematic review provides a thorough overview of the literature on neuroimaging biomarkers and their significance in predicting outcomes following stroke recovery.

### 2.6 Quality appraisal

Critical Appraisal Skills Program (CASP) checklist was used to assess the quality and reliability of randomized controlled trials. Timeline development, measurement mistakes, blinding, subpar assessments, selective clinical quality, and other biases were all assessed, with low, high, and unknown risks of bias being categorized as appropriate.

A 10‐item checklist was used to evaluate the articles' systematic review (see Table 2). Several aspects were considered, such as the technique's validity, the results' efficacy, and the study approach's dependability. The processes' methodological validity was examined and questioned. The results' small sample size and incisiveness were considered while evaluating external validity; those with notably poor accuracy were disregarded.

**Table 2:** Demonstrating the validity of the selected studies (*N* = 11).

Number	References	Was there a clear statement of the aims of the research?	Is there an appropriate qualitative methodology?	Was the research design appropriate to address the aims of the research?	Was the recruitment strategy appropriate to the aims of the research?	Was the data collected in a way that addressed the research issue?	Has the relationship between researcher and participants been adequately considered?	Have ethical issues been taken into consideration?	Was the data analysis sufficiently rigorous?	Is there a clear statement of the findings?	Is the research valuable?	Total
1	[28]	Yes	Yes	Yes	Yes	Yes	Yes	No	Yes	Yes	Yes	9
2	[29]	Yes	Yes	Yes	Yes	Yes	Yes	Yes	Unclear	Yes	Yes	9
3	[30]	Yes	Yes	Yes	Yes	Yes	Yes	Yes	Yes	Yes	Unclear	9
4	[31]	Yes	Yes	Yes	Yes	Yes	No	Yes	Yes	Yes	Yes	9
5	[32]	Yes	Yes	Yes	Yes	Yes	Yes	Yes	Yes	Yes	Yes	10
6	[33]	Yes	Yes	Yes	Yes	Yes	Yes	Yes	Yes	Yes	Unclear	9
7	[34]	Yes	Yes	Yes	Yes	Yes	Yes	Yes	Unclear	Yes	Unclear	8
8	[35]	Yes	Yes	Yes	Yes	Yes	Yes	Yes	Yes	Yes	Unclear	9
9	[26]	Yes	Yes	Yes	Yes	Yes	Yes	Yes	Yes	Yes	Yes	10
10	[36]	Yes	Yes	Yes	Yes	Yes	Yes	Yes	Yes	Yes	Yes	10
11	[37]	Yes	Yes	Yes	Yes	Yes	Yes	Yes	Yes	Yes	Yes	10

### 2.7 Evidence worksheet scoring

Studies scoring six or higher are classified as evidence level 1 (6–8 being good, 9–10 being outstanding), while studies scoring four or less are classified as level of evidence 2 (4–5 being acceptable, less than 4 being bad).

### 2.8 Data extraction

Eleven articles were scanned for data for this evaluation using the CRD (2009) standards. The information extraction table contains the study's methodology, stroke conditions, clinical parameters gathered by the researcher, and biomarkers used at the time of prediction. When investigations were discussed in published papers, pertinent information was collected from each publication.

### 2.9 Data items

- Study author.
- Study type.
- Types of biomarkers associated with the stroke outcome.

### 2.10 Information sources

1. PubMed or Medline (2010–2024)
2. Cochrane Reviews (2010–2024).

## 3 RESULTS

**Figure 1:**
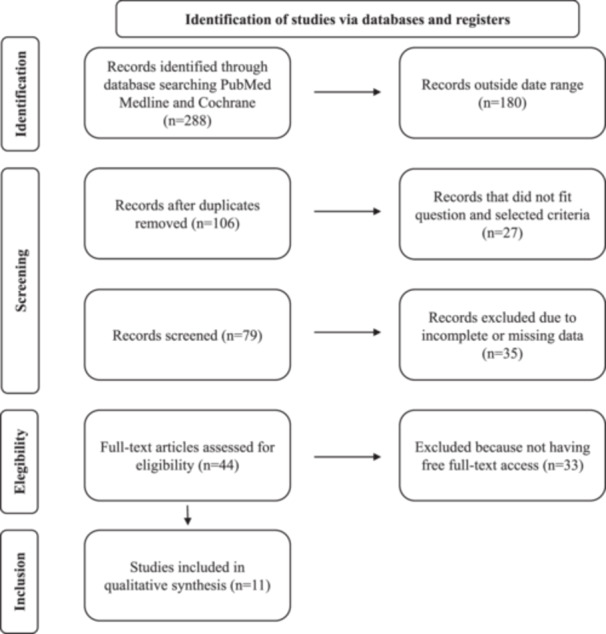
Flow diagram showing the information of every stage of the systematic review.

The combination of keywords in the database searches yielded 286 documents, as illustrated in Figure 1. One hundred and eighty articles were screened for eligibility, and duplicates were then eliminated. After the screening process, 11 articles were included in the review (see Table 3). Figure 1 illustrate the entire selection process for each of the pertinent procedures.

**Table 3:** Summary of the included studies.

Number	References	Study type	Objective	Conclusion
1	[28]	A prospective study	To evaluate the association between volumetric imaging markers in acute stroke and patient‐reported outcome measures	Higher White matter hyperintensity was linked to worse outcomes, although higher estimated brain volume was defensive, according to neuroimaging biomarkers that were strongly correlated with patient‐reported outcome measures. Improved knowledge of poststroke outcomes is made possible by routine assessment that incorporates neuroimaging analysis and patient‐reported outcome measures.
2	[29]	Review	To provide an overview of the approaches currently employed in atlas‐based voxel neuroimaging feature‐based motor outcome prediction research	Validating imaging predictors, advancing methodological approaches, and establishing reporting guidelines are all necessary for neuroimaging biomarker development for the prediction of poststroke motor outcomes
3	[30]	Review	To conduct a comprehensive assessment of the scope of research on neuroimaging's applicability and usage as a predictive biomarker for cognitive recovery following a stroke	Potential biomarkers for cognitive recovery following ischemic stroke include Diffusion Imaging and Functional MRI employing resting‐state functional connectivity, while more accurate predicting methods are required. Though there is currently no neuroimaging technique that can be used as a biomarker, this research points to a therapeutically important role for neuroimaging in predicting cognitive recovery following a stroke.
4	[31]	Review	To evaluate the clinical application that may effectively predict the functional results of stroke survivors, substantially enhancing the effectiveness of stroke patients' rehabilitation	To improve the predictive ability of stroke prognostic models, a blood‐derived multibiomarker is suggested as a suitable method in addition to neuroimaging and neurophysiological biomarkers
5	[32]	Observational study	To assess the relationship between severe upper limb impairment following a stroke and imaging biomarkers beyond the corticospinal tract by analyzing the white matter microstructure in the corpus callosum	The microstructure of the corpus callosum could be a useful biomarker for the fate of severe upper limb loss following a stroke, which could improve our capacity to forecast recovery in patients with severe motor impairment
6	[33]	Research article	Examine the function of neuroimaging and biomarkers in stroke risk assessment for the prevention of cardiovascular disease	It has been confirmed that neuroimaging and other biomarkers can be used to stratify an older person's risk of a brain hemorrhage. Choosing the right anticoagulant or closing the left atrial appendage to avoid stroke in atrial fibrillation patients requires a detailed understanding of these indicators.
7	[34]	An observational, prospective study	To assess the efficacy of clinical features, serum biomarkers, stroke cause, and neuroimaging as predictors of upper limb functionality 12 weeks following stroke	Poor upper limb functionality linked to advanced age. Improved upper limb functioning is predicted by an ASPECTS score of ≥8 within 24 h and an S100β protein of <140.5 ng/L at 48 h; poorer upper limb functioning is predicted by advanced age 12 weeks after stroke.
8	[35]	Review	To estimate a patient's motor recovery and outcome with reliability by advancements in biomarkers	It is concluded that indicators of corticomotor anatomy and function, both neurophysiological and neuroimaging, may be beneficial in predicting motor outcomes and long‐term recovery following a stroke
9	[26]	A systematic review	To evaluate the data and identify the neurological biomarker or biomarkers, which are used to predict motor recovery and satisfy the demanding evidence quality standards	The combined biomarker categories and conventional structural MRI were rated as having “high” overall evidence quality. To determine which neuroimaging biomarker is the most effective indicator of recovery following stroke, more high‐quality research is required. Lastly, more modern techniques and statistical approaches should be added to the 25‐year‐old methodological quality tool used here.
10	[36]	Review	The purpose of this review is to gather evidence that suggests the use of neuroimaging data to predict recovery and long‐term outcomes could prove beneficial	It can be difficult to predict long‐term functional results after an ischemic stroke; nevertheless, imaging techniques throughout the hyperacute and subacute phases offer benefits over clinical prediction factors. This data may be used in the future to direct acute and rehabilitative therapy as well as offer useful prognostic information when coupled with clinical data.
11	[37]	A systematic review	This study aims to evaluate the methodological quality of imaging studies regarding, recovery within 6 months poststroke and to find patterns in the relationship between functional upper limb recovery and task‐related brain activity patterns	Trends that can be identified despite variability and shortcomings in methodology. Statistically significant correlations with recovery, however, are inconsistent. To determine the fMRI and PET validity about upper limb recovery following a stroke, future research will need to precisely measure limb dexterity, control for the amount of white matter damage, and find ways to adjust for variations in perfusion.

Abbreviations: ASPECTS, Alberta Stroke Program Early Computed Tomography Score; fMRI, functional magnetic resonance imaging; MRI, magnetic resonance imaging; PET, positron emisión tomography.

## 4 DISCUSSION

### 4.1 Neuroimaging biomarkers

Many neuroimaging biomarkers, encompassing functional and structural aspects, have been explored in stroke patients.^37^ Structural integrity assessments using diffusion‐weighted and T1‐weighted MRI allow the evaluation of the cortex and white matter pathways. For instance, diffusion‐weighted MRI facilitates the examination of white matter damage and microstructural organization, with metrics such as axial diffusivity and fractional anisotropy revealing associations with motor function.^38^

In a study conducted within 12 h of stroke onset, diffusion‐weighted images were obtained from 60 patients. Raters assessed the impact on the precentral gyrus and occipital part of the brain, revealing that destruction to the posterior limb was the most reliable predictor of outcomes at 90 days.^39^ Subsequent investigation determined that fractional anisotropy and corticospinal tract (CST) asymmetry at the brainstem were associated with worse motor outcomes 2 years poststroke.^40^

However, limitations arise from using gross measures of proximal movement in motor outcome classifications in some studies. The Fugl‐Meyer Assessment for Upper Extremity score and acute lesion load on the ipsilesional CST were negatively correlated 3 months poststroke.^41^, ^42^ Despite predicting coarse motor outcomes, these white matter integrity biomarkers from neuroimaging could prove valuable for tailoring rehabilitation plans and selecting participants for clinical studies.^41^, ^42^

White matter integrity biomarkers from neuroimaging may also be helpful indicators of motor recovery. Lower recovery from an impairment compared to the extent to which it was severe in the initial case is the consequence of more impairment to the CST caused by stroke lesions.^43^, ^44^ According to research, in 23 of 27 participants, more significant fractional anisotropy (FA) impairments in the CST can recognize individuals who will not recover proportionately from upper‐limb motor impairment. Moreover, FA asymmetry at the subsequent limbs of the inner capsules may differentiate motor evoked potential (MEP)‐negative patients into those who would essentially not recover voluntary motion in upper limbs and those who will only partially recover.^44^ To validate these initial results, larger patient groups are necessary. Brain activity and connectivity during rest or motor task performance can be obtained with functional MRI. Overall, improved motor performance during scanning is associated with more typical patterns (such as those observed among healthy controls) of task‐related brain activity and resting‐state functional connectivity.^37^, ^45^, ^46^, ^47^

Functional MRI measurements may also predict upper‐limb motor results. Two studies,^48^, ^49^ both with 21 patients, found task‐related brain activity patterns that could be used to predict future motor results. Another investigation, conducted within a week after a stroke, involved individuals with minimal‐to‐serious upper‐limb disability who had their paretic upper limbs scanned while performing a hand‐grip activity.^49^ Four to six months after the stroke, patients were categorized into groups with excellent or poor motor efficiency using a median split. This was determined by combining the Action Research Arm Test results and grip capability to generate a composite score. Patients experiencing favorable motor outcomes during the acute phase of a stroke showed more activity in their contralesional cerebellum, ipsilesional premotor cortex, and ipsilesional primary motor cortex than patients with adverse motor outcomes.^49^ For a comprehensive overview of neuroimaging techniques in stroke research, please see Table 4.

**Table 4:** Neuroimaging techniques.

Neuroimaging biomarkers	Description
Diffusion‐weighted MRI	Assess structural integrity, examine white matter damage and microstructural organization
T1‐weighted MRI	Evaluate cortical and white matter pathways
Functional MRI	Measure brain activity and connectivity during rest or task performance
TMS	Assess corticomotor function, predict motor outcomes

Abbreviations: MRI, magnetic resonance imaging; TMS, transcranial magnetic stimulation.

An additional study^48^ discovered that patients were scanned while their paretic wrist was in a passive position 1 month after a moderate‐to‐severe stroke. A multivariable regression model explained 87% of the variability in the motor as a whole 6‐month Fugl‐Meyer score following a stroke. This model included baseline overall FMA‐UE motor score (up to 100 points) and task‐related cortical activity. The variance that had been explained increased to 96% once the baseline overall FMA‐UE motor score was eliminated. According to these researchers,^48^, ^49^ patterns of active and passive task‐associated brain activity detected by functional magnetic resonance imaging may predict comparable outcomes and possibly higher prediction values for motor outcomes than clinical scores. However, research with small sample sizes places limitations on them, and larger patient cohorts are needed to validate these biomarkers.

Neuroimaging biomarkers can examine the composition and operation of the more extensive sensorimotor network, which includes the cerebellum, sensory cortex, and nonprimary motor cortex. The results obtained from combining the neuroimaging biomarkers discussed in this section validate the pivotal function of the ipsilesional motor cortex and CST in restoring motor function following a stroke. Fractional anisotropy and lesion load, two metrics that indicate the degree of damage caused by a stroke to the ipsilesional descending white matter pathways, may have threshold values that could be utilized to forecast outcomes for specific individuals. Robust functional neuroimaging biomarkers will require further study with larger patient cohorts.^50^

### 4.2 Implications for clinical practice

Studies have shown that stroke survivors commonly experience cognitive deficits, which can range from mild impairments to severe dementia, affecting various domains such as memory, attention, and executive function.^51^ It has also been demonstrated that aphasia, a language disorder characterized by difficulty in speaking, understanding, reading, and writing, occurs in a significant proportion of stroke survivors.^52^ Assessing speech impairment as an outcome measure poststroke is crucial for developing targeted interventions and rehabilitation strategies to improve communication outcomes. Predicting a patient's functional outcomes and recovery from motor impairment is a challenging but essential part of rehabilitation and discharge planning.^53^ Patients with initially significant upper‐limb disability may benefit from differentiating between those who will recover their motor skills proportionately and those who will not. This can be done by looking at their MEP status. Finding patients who are significantly disabled and have the potential for proportionate improvement may make it possible to provide them with suitable rehabilitation that advances their functional recovery over time. It is less therapeutically valuable to predict the impairment score of a patient, for example, based on the FMA‐UE score (ranging from 26 to 30). Compared to predicting their motor function outcomes, the latter result indicates the patient's independence in daily activities.^54^, ^55^ By merging biomarkers, predictions could become more accurate. Multivariable regression models incorporating biomarker, clinical, and demographic data have been employed in most investigations to account for variations in motor recovery or outcomes for patient groups. However, when predicting specific patients in clinical practice, the regression equations produced are not very helpful.^26^, ^56^, ^57^

To date, only one method combines biomarkers in the initial days following a stroke to predict outcomes for specific patients. By sequentially combining biomarkers, the Predict Recovery Potential (PREP) algorithm predicts functional consequences for the upper limb.^58^ Neurophysiological and neuroimaging measurements come first to determine a prognosis, followed by clinical measurements. The PREP algorithm mitigates several disadvantages of establishing prediction on specific clinical or measurement biomarkers. Clinical evaluation does not predict recovery in patients with initially severe motor impairment.^42^, ^43^, ^44^, ^50^

To determine the motor‐evoked potential (MEP) condition of patients with significant upper limb damage at their initial visit. The algorithm employs transcranial magnetic stimulation. Patients who test positive for MEP have a good chance of recovering and achieving a functional outcome. Although the lack of MEPs does not always imply a bad result, their absence does suggest a positive outcome.^35^, ^50^ To resolve this ambiguity, the algorithm studies the structural integrity of all sensory pathways that pass through the posterior limbs of the patients' internal capsules using diffusion‐weighted MRI for patients who are MEP negative. Patients identified with negative MEP and expected to improve are distinguished from patients by the extent of damage associated with these methods. About half of patients suffering from stroke require diffusion‐weighted MRI because they are MEP negative, and transcranial magnetic stimulation is necessary for one‐third of the patients.^35^, ^59^ This research suggests that biomarkers may be helpful in clinical settings for forecasting motor function results, while further research is required in various rehabilitation contexts.

Residual motor impairment is a common outcome after proportional rehabilitation for most patients. Therefore, the primary objective of treatment may be to assist patients in living with and compensating for their disability to enhance their functional outcomes. Research has indicated that physical therapy assists in restoring motor function following a stroke.^60^, ^61^ However, additional high‐quality data supporting frequently employed upper‐limb therapies is required.

A meta‐regression assessment^62^ of data from 30 studies involving 1750 patients supports the finding that higher treatment dosages are more advantageous, regardless of when rehabilitation started. Scheduled treatment time was found to have a small but substantial positive correlation with motor outcome. The Action Research Arm Test results demonstrate the recovery of upper‐limb functioning did not correlate with the therapeutic dosage, as determined by task repetitions, in a later trial (*n* = 85) that examined stroke patients in the chronic stage.

Lang et al.^63^ emphasizes the importance of beginning stroke therapy as soon as possible because the first few days and weeks following a stroke are essential for neurological recovery.^64^ A recent study that used a mouse stroke model demonstrated this critical interval. The authors discovered that partial task performance recovery occurred when the poststroke practice of a proficient reach‐to‐grasp task was delayed by 7 days. When the same hemisphere was the site of a second stroke, practice resumed a day later, and task performance returned to prestroke levels in total. This mouse study shows a brief window of advantageous physiologic circumstances following a stroke, which facilitates a positive response to training.^65^ Increased therapeutic dosages seem to work in combination with these advantageous biological circumstances to promote restoration of motor functions, yet not a recovery from disability that is commensurate. Improving proportionate rehabilitation from impairment may necessitate therapies with mechanisms of action that are significantly different from those used in present therapy procedures.

### 4.3 Implications for clinical research

In clinical trials of poststroke motor rehabilitation, biomarkers may be helpful for patient categorization and selection. This question was addressed by reviewing rehabilitation trials with motor primary outcomes. Eight multicenter, assessor‐blind trials^66^, ^67^, ^68^, ^69^, ^70^, ^71^, ^72^, ^73^ analyzing data from at least 100 patients have been conducted since 2011 on physical therapy initiated at the acute and subacute stages of stroke. The strengths of these studies include reports on dosages of frequently used medications,^67^, ^68^, ^71^, ^72^ frequent clinical measurements taken on a strictly regulated schedule, and the implementation of a dose‐matched, active control intervention.^67^, ^71^, ^72^

Studies examining the impact of varying intervention durations on the fundamental processes of motor rehabilitation are limited by the lack of subsequent measurements^66^ and the enrollment of patients for 3 months^71^ or 6 months^68^ following a stroke. According to the two following trials^66^, ^70^ there was no usual‐care control group; therefore, they could not identify any benefit from the treatment compared to standard therapeutic procedures. None of these investigations^66^, ^67^, ^68^, ^69^, ^70^, ^71^, ^72^, ^73^ discovered any variations in rehabilitation or outcomes between the group receiving treatment as well as the control groups, even though all of them demonstrated improvements in patients' motor ability. It's possible that the treatment and control interventions appeared comparable because all of these trials evaluated interventions that were modifications of established therapeutic methods.^74^ Second, no corticomotor function or structure biomarkers were employed in these investigations to determine which individuals to include. Because of this, even though the groups were matched based on baseline clinical scores, there may have been differences in the patients' ability to respond to the interventions between the treatment and control groups. A considerable cohort enrollment will likely provide balanced groupings concerning important biomarkers and predictors. However, it does not guarantee that individuals for whom the intervention is ineffective will be excluded.

The use of neuroimaging biomarkers in patient selection could enhance the trial's statistical power to identify intervention effects by enabling patients to be chosen based on their tendency to respond to the biological mechanism of action of the intervention.^50^

## 5 CONCLUSION

Stroke is a prominent cause of long‐term adult impairment globally and a significant global health issue. The utilization of advanced neuroimaging techniques and exceptionally successful reperfusion therapies throughout the poststroke period results in better care and a decreased chance of death for stroke patients. These interventions contribute to the diagnosis of stroke and also assist in the improvement of any neurological impairment caused by the stroke. Stroke survivors do not, however, have widespread access to diagnostic or treatment approaches, particularly in underdeveloped nations. Additionally, it should be considered that the neurological prognosis following a stroke seems to vary significantly. There are two types: mild, nondisabling, and severe, which can cause death or permanent impairment. Transcranial magnetic stimulation's principal drawback can be avoided by neuroimaging biomarkers that evaluate the composition and functionality of the more extensive sensorimotor network, including the cerebellum and sensory or somatosensory cortex, which can prevent transcranial magnetic stimulation's principal drawback. The results of this study substantiate the significance of neuroimaging biomarkers in recovering motor function after a stroke.

## AUTHOR CONTRIBUTIONS

**Elizabeth Gaviria**: Conceptualization; writing—original draft; visualization; methodology; formal analysis. **Awab Hamid Eltayeb Hamid**: Writing—review & editing; formal analysis.

## CONFLICT OF INTEREST STATEMENT

The authors declare no conflict of interest.

## TRANSPARENCY STATEMENT

The lead author Awab Hamid Eltayeb Hamid affirms that this manuscript is an honest, accurate, and transparent account of the study being reported; that no important aspects of the study have been omitted; and that any discrepancies from the study as planned (and, if relevant, registered) have been explained.

## Data Availability

Data sharing is not applicable as no new data were generated or analyzed in this review.
